# Editorial: Parasitism: the good, the bad and the ugly

**DOI:** 10.3389/fvets.2023.1304206

**Published:** 2023-10-17

**Authors:** Marco A. Juarez-Estrada, Danielle Graham, Xochitl Hernandez-Velasco, Guillermo Tellez-Isaias

**Affiliations:** ^1^Department of Medicine and Zootechnics of Birds, College of Veterinary Medicine and Zootechnics, National Autonomous University of Mexico, Mexico City, Mexico; ^2^Division of Agriculture, Department of Poultry Science, University of Arkansas, Fayetteville, AR, United States

**Keywords:** protozoa, helminths, ectoparasites, parasite vaccines, mutualism, inflammation

Parasitism is a tight association between species in which one organism, the parasite, lives on or inside the host, causing it harm, and is structurally adapted to this way of life (1). Until the twenty-first century, parasitism was studied by parasitologists, rather than ecologists or evolutionary biologists. Today, parasitism is a major element of evolutionary ecology, as nearly all free-living animals, are hosts to at least one parasite species (2, 3). Since it is in the parasite's evolutionary interest for its host to flourish, long-term coevolution can lead to a stable relationship bordering on mutualism (1, 4). According to Lynn Margulis, when resources are scarce, natural selection moves relationships from parasitism to mutualism, as was brilliantly illustrated in Margulis' endosymbiosis theory, where eukaryotic mitochondria and chloroplasts descended from formerly free-living prokaryotes (5).

While worm parasitism clearly affects animals, it may also reduce the prevalence and severity of autoimmune diseases in animal hosts, including humans (6). Helminth infections might safeguard hosts from immune-mediated illnesses like allergies and autoimmune disease, as well as suppress detrimental inflammatory host's reaction. In this symbiotic relationship, the helminth, and the host benefit from each other's presence (7–9). The boundary between mutualism, symbiosis, and pathological parasitism is narrow and is frequently overlapping without a theory to clearly explain this relationship between the parasite, host, and ecological niche (10–12). Parasites are frequently seen as dangerous and primarily viewed as harmful to the host (3, 13). However, removing them entirely would be detrimental to the host, the environment, and the parasites themselves (3). Complex natural ecosystems with diverse species and their interactions are the result of a long evolutionary history (12, 14). Parasites make up half of life's diversity and play vital ecological roles (3, 15). Parasites allow for inter-species DNA exchange, allowing for evolutionary change (16). To complete their life cycles, many parasites rely on predator-prey or other stable ecological interactions (12, 17). At individual and species levels, hosts have developed adaptations to their coevolving parasites (10, 11, 15, 18). Often, the expression of normal behavior or the development of a healthy immune system depends on parasite infections (8). In instances where parasites are not present, hosts may occasionally exhibit phenotypes that are less than ideal (14). Conserving parasite species is supported by their evolutionary heritage and potential, their role in ecosystem services, and their value as indicators of ecosystem quality (3, 19). Parasites imply a healthy ecology (20). Researchers have highlighted the functional significance of parasites in preserving the health of individual hosts, host populations, and entire ecosystems in order to raise awareness of parasite conservation (3, 14). For individual hosts, parasites impose energetic demands that reduce growth, fecundity, and survival (21, 22), but these apparent negative effects on host fitness may be compensated by positive consequences, such as improving normal host immune system function (8, 9, 23), regulating their host's microbiome (7, 8, 24–26), inducing specific interaction between gut microbiota and host immune responses (27–29), and providing protection against pollutants such as heavy metals (20, 30, 31). Even though parasites decrease host fitness, they could potentially maintain host population viability by controlling host population size (18, 32, 33), and increasing genetic variety while avoiding the detrimental effects of inbreeding (21). Parasites can act as keystone species, allowing competing species to coexist while lowering the dominance of superior competitors (17). Parasites may mediate predatory and competitive interactions among free-living species at higher levels of organization, adjusting community structure and diversity, food web complexity, and energy flow throughout the entire ecosystem (20, 34–36). While parasites typically remain absent from food web representations, they consistently hold the top position (15).

With 220 million people affected annually by malaria, measures such as prophylaxis, eradicating mosquito vectors using pesticides, and developing a malaria vaccine, have been attempted to stop malaria transmission (37). Nonetheless, medication resistance, mosquito pesticide resistance, and vaccination failures due to parasite mutations or parasite immune manipulation have all been issues (38–40). Similar problems have been observed with other economically significant groups of parasites that cause disease in livestock, poultry, and domestic animals, such as protozoa, helminths, and ectoparasites (41, 42).

Due to their potential benefits in numerous ecosystems, parasites should not be eliminated from Earth's environments (3). Nonetheless, some parasites do represent a serious threat to human and animal health; therefore, we must find an appropriate equilibrium between parasite burden, human and animal fitness, and ecosystem quality, all while adhering to the current one health concept (14, 27, 33, 43–46). To achieve this equilibrium, parasite managements must rely on suitable treatment, prevention and control strategies, and their appropriate implementation at field (3, 29, 38, 47). Studies on parasites management can serve as a link between health and environmental sciences, enabling accomplish the objective of the one health concept through an interdisciplinary and holistic approach (14, 44, 48).

Several issues regarding holistic approaches to parasite management have aroused the interest of scientific community, with resulting in several investigations and publications to answer if parasites are good, bad, or ugly for human's and animal's health. This Research Topic contains all various initiatives to harness knowledge, such as the following:

1. Genetic studies for both ecto- and endo parasites. Generated mitochondrial genome data provided further flea taxonomic and epidemiological studies (Liu et al.). Genetic analysis of *Fasciola hepatica*, demonstrated population structure in South America not previously realized using nuclear and mitochondrial markers (Garcia-Corredor et al.).

2. Innovative lab procedures. Currently, it is feasible to use harmless methods for preserving avian *Eimeria* sp. sporulated oocysts for challenge testing or live coccidiosis vaccines (Laverty et al.). Isolates of live-attenuated *Histomonas meleagridis* were successfully obtained by serial passaging in a lab setting (Beer et al.). These attenuated vaccine strains proved to be immunoprotective against challenge with virulent *H. meleagridis*. However, commercial application of these attenuated vaccines is not currently feasible from a production, labor, and economic standpoint. Nowdays, the identification of *E. tenella* antigens through novel immunological approaches is crucial for the development of successful genetically engineered vaccines (Juárez-Estrada et al.). Lab procedures indicates that Ferritin 2 has emerged as a promising antigen for a universal vaccination against avian mites (Win et al.).

3. Bibliometric analysis for screening assistance. Bibliometric data provides insight for the selection and evolution of anticoccidial drugs and, contributes to understand anticoccidial medication utilization trends (Kandeel et al.).

4. Experimental infection models are necessary to mimic complex multi-factorial interactions. An experimental mixed avian *Eimeria* sp. challenge model that particularly evaluates performance, intestinal permeability, and dysbiosis is suitable to analyse different strategies for avian coccidiosis control (Graham et al.). An experimental model revealed that horizontal transmission of *H. meleagridis* could vary depending on the pathogenicity of the challenge strain and Turkey diet composition (Barros et al.). Turkeys experimentally vaccinated with a candidate *E. meleagrimitis* strain with or without amprolium intervention showed altered ileal and cecal microbiome (Trujillo-Peralta et al.). In an experimental model of priming/challenge, an *E. meleagrimitis* strain candidate vaccine caused a moderate infection that induced protective immunity (Trujillo-Peralta et al.). Coccidiosis infection in lambs impacts rumen fermentation in a way that is independent of plane nutrition, but this effect does not translate to performance response (Sujani et al.).

5. Alternatives to antibiotics. As demand rises to decrease (eliminate) antibiotic use in poultry; natural resources such as *Yucca schidigera* and *Trigonella foenum* are promising coccidiosis control options (Benarbia et al.). Soybean lecithin has also been linked to increased resilience in several animal species to physical and chemical stressors (Wee et al.).

Novel research on parasitology contributes to identify new prevention and control measures that we need now more than ever front to current climate changing (43, 49, 50).

## Author contributions

MJ-E: Conceptualization, Data curation, Writing—original draft, Writing—review and editing. GT-I: Conceptualization, Data curation, Funding acquisition, Writing—original draft, Writing—review and editing. DG: Conceptualization, Data curation, Writing—original draft, Writing—review and editing. XH-V: Data curation, Visualization, Writing—review and editing.

## References

[1] Rózsa L Garay J. Definitions of parasitism, considering its potentially opposing effects at different levels of hierarchical organization. Parasitol. (2023) 150:761–68. 10.1017/S0031182023000598 37458178 PMC10478066

[2] Santiago MFM King KC Drew GC. Interactions between insect vectors and plant pathogens span the parasitism–mutualism continuum. Biol Lett. (2023) 19:20220453. 10.1098/rsbl.2022.0453 36883313 PMC9993222

[3] Lymbery AJ Smit NJ. Conservation of parasites: a primer. Int J Parasitol Parasites Wildl. (2023) 21:255–63. 10.1016/j.ijppaw.2023.07.001 37483309 PMC10359719

[4] Hasik AZ de Angeli Dutra D Doherty JF Duffy MA Poulin R Siepielski AM. Resetting our expectations for parasites and their effects on species interactions: a meta-analysis. Ecol Lett. (2023) 26:184–99. 10.1111/ele.14139 36335559 PMC10099232

[5] López-García P Moreira D. The symbiotic origin of the eukaryotic cell. C R Biol. (2023) 346:55–73. 10.5802/crbiol.118 37254790

[6] Abu-Shakra M Shoenfeld Y. For debate parasitic infection and autoimmunity. Autoimmunity. (1991) 9:337–44. 10.3109/08916939108997136 1954314

[7] Loke P Lim YAL. Helminths and the microbiota: parts of the hygiene hypothesis. Parasite Immunol. (2015) 37:314–23. 10.1111/pim.12193 25869420 PMC4428757

[8] Ramanan D Bowcutt R Lee SC Tang MS Kurtz ZD Ding Y. Helminth infection promotes colonization resistance via type 2 immunity. Science. (2016) 352:608–12. 10.1126/science.aaf3229 27080105 PMC4905769

[9] Atagozli T Elliott DE Ince MN. Helminth lessons in inflammatory bowel diseases (IBD). Biomedicine. (2023) 11:1200. 10.3390/biomedicines11041200 37189818 PMC10135676

[10] Taraschewski H. Hosts and parasites as aliens. J Helminthol. (2006) 80:99–128. 10.1079/JOH2006364 16768855

[11] Lymbery AJ Morine M Kanani HG Beatty SJ Morgan DL. Co-invaders: the effects of alien parasites on native hosts. Int J Parasitol Parasites Wildl. (2014) 3:171–77. 10.1016/j.ijppaw.2014.04.002 25180161 PMC4145144

[12] Best A Ashby B. How do fluctuating ecological dynamics impact the evolution of hosts and parasites? Phil Trans R Soc B. (2023) 378:20220006. 10.1098/rstb.2022.0006 36744565 PMC9900711

[13] Vilcinskas A. Pathogens as biological weapons of invasive species. PLoS Pathog. (2015) 11:e1004714. 10.1371/journal.ppat.1004714 25856550 PMC4391917

[14] Selbach C Mouritsen KN Poulin R Sures B Smit NJ. Bridging the gap: aquatic parasites in the One Health concept. Trends Parasitol. (2022) 38:109–11. 10.1016/j.pt.2021.10.007 34863638

[15] Jephcott TG Sime-Ngando T Gleason FH Macarthur DJ. Host–parasite interactions in food webs: Diversity, stability, and coevolution. Food Webs. (2016) 6:1–8. 10.1016/j.fooweb.2015.12.001

[16] Combes C. Parasitism: The Ecology and Evolution of Intimate Interactions. Chicago: University of Chicago Press Chicago, USA. (2001).

[17] Ramesh A Hall SR. Niche theory for within-host parasite dynamics: Analogies to food web modules via feedback loops. Ecol Lett. (2023) 26:351–68. 10.1111/ele.14142 36632705

[18] Papkou A Gokhale CS Traulsen A Schulenburg H. Host–parasite coevolution: why changing population size matters. Zoology. (2016) 119:330–38. 10.1016/j.zool.2016.02.001 27161157

[19] Almeida D Cruz A Llinares C Torralva M Lantero E Fletcher DH. Fish morphological and parasitological traits as ecological indicators of habitat quality in a Mediterranean coastal lagoon. Aquatic Conserv: Mar Freshw Ecosyst. (2023) 2023:1–16. 10.1002/aqc.3996

[20] Marcogliese DJ. Parasites of the superorganism: are they indicators of ecosystem health? Inter J Parasitol. (2005) 35:705–16. 10.1016/j.ijpara.2005.01.015 15925594

[21] Coltman DW Pilkington JG Smith JA Pemberton JM. Parasite-mediated selection against inbred soay sheep in a free-living, island population. Evol. (1999) 53:1259–67. 10.2307/2640828 28565537

[22] Marcogliese DJ. Parasites: Small players with crucial roles in the ecological theater. Ecohealth. (2004) 1:151–64. 10.1007/s10393-004-0028-3

[23] Sorci G Guivier E Lippens C Faivre B. Microbes, Parasites and Immune Diseases. In: Alvergne A Jenkinson C Faurie C, editors. Evolutionary Thinking in Medicine: From Research to Policy and Practice. Cham: Springer International Publishing. (2016) p. 211–23.

[24] Giacomin P Agha Z Loukas A. Helminths and intestinal flora team up to improve gut health. Trends Parasitol. (2016) 32:664–66. 10.1016/j.pt.2016.05.006 27234811

[25] Partida-Rodríguez O Serrano-Vázquez A Nieves-Ramírez ME Moran P Rojas L Portillo T. Human intestinal microbiota: Interaction between parasites and the host immune response. Arch Med Res. (2017) 48:690–700. 10.1016/j.arcmed.2017.11.015 29290328

[26] Llewellyn MS Leadbeater S Garcia C Sylvain FE Custodio M Ang KP. Parasitism perturbs the mucosal microbiome of Atlantic Salmon. Sci Rep. (2017) 7:43465. 10.1038/srep43465 28266549 PMC5339869

[27] Mutapi F. The gut microbiome in the helminth infected host. Trends Parasitol. (2015) 31:405–06. 10.1016/j.pt.2015.06.003 26142923

[28] Li C Liu Y Liu X Bai X Jin X Xu F. The gut microbiota contributes to changes in the host immune response induced by *Trichinella spiralis*. PLoS Negl Trop Dis. (2023) 17:e0011479. 10.1371/journal.pntd.0011479 37585413 PMC10431649

[29] Boisseau M Dhorne-Pollet S Bars-Cortina D Courtot É Serreau D Annonay G. Species interactions, stability, and resilience of the gut microbiota - Helminth assemblage in horses. iScience. (2023) 26:106044. 10.1016/j.isci.2023.106044 36818309 PMC9929684

[30] Sánchez MI Pons I Martínez-Haro M Taggart MA Lenormand T Green AJ. When parasites are good for health: Cestode parasitism increases resistance to arsenic in brine shrimps. PLoS Pathog. (2016) 12:e1005459. 10.1371/journal.ppat.1005459 26938743 PMC4777290

[31] Keke UN Mgbemena AS Arimoro FO Omalu ICJ. Biomonitoring of effects and accumulations of heavy metals insults using some helminth parasites of fish as bio-indicators in an Afrotropical stream. Front Environ Sci. (2020) 2:576080. 10.3389/fenvs.2020.576080

[32] Hudson PJ Dobson AP Newborn D. Prevention of population cycles by parasite removal. Science. (1998) 282:2256–58. 10.1126/science.282.5397.2256 9856948

[33] Scott ME. Helminth-host-environment interactions: Looking down from the tip of the iceberg. J Helminthol. (2023) 97:e59. 10.1017/S0022149X23000433 37486085

[34] Hudson PJ Dobson AP Lafferty KD. Is a healthy ecosystem one that is rich in parasites? Trends Ecol Evol. (2006) 21:381–85. 10.1016/j.tree.2006.04.007 16713014

[35] Dunn AM Hatcher MJ. Parasites and biological invasions: parallels, interactions, and control. Trends Parasitol. (2015) 31:189–99. 10.1016/j.pt.2014.12.003 25613560

[36] Vannatta JT Minchella DJ. Parasites and their impact on ecosystem nutrient cycling. Trend Parasitol. (2018) 34:452–55. 10.1016/j.pt.2018.02.007 29526401

[37] El Moustapha I Ouldabdallahi Moukah M Ould Ahmedou Salem MS Brahim K Briolant S Basco L. Malaria prevalence in Mauritania: a systematic review and meta-analysis. Malar. J. (2023) 22:146. 10.1186/s12936-023-04569-4 37131226 PMC10152621

[38] Orwa T Mbogo R Luboobi L. Mathematical model for the in-host malaria dynamics subject to malaria vaccines. Lett Biomath. (2018) 5:22–251. 10.30707/LiB5.1Orwa

[39] Aidoo EK Aboagye FT Botchway FA Osei-Adjei G Appiah M Duku-Takyi R. Reactive case detection strategy for malaria control and elimination: a 12 year systematic review and meta-analysis from 25 malaria-endemic countries. Trop Med Inf Dis. (2023) 8:180. 10.3390/tropicalmed8030180 36977181 PMC10058581

[40] Miyauchi E Shimokawa C Steimle A Desai MS Ohno H. The impact of the gut microbiome on extra-intestinal autoimmune diseases. Nat Rev Immunol. (2023) 23:9–23. 10.1038/s41577-022-00727-y 35534624

[41] Blake DP Tomley FM. Securing poultry production from the ever-present *Eimeria* challenge. Trends Parasitol. (2014) 30:12–9. 10.1016/j.pt.2013.10.003 24238797

[42] Naz S Abbasi SW Cavezza S Jaiswal AK Azevedo V. Parasite Control Strategies: Immunoprophylaxis. In: Rizwan HM Sajid MS, editors. Parasitism and Parasitic Control in Animals. Oxfordshire: CABI. (2023) 231–47. 10.1079/9781800621893.0015

[43] Iacovino A Randazzo L. Climate change and food security in the One Health perspective. BioLaw J. (2023) 2:293–314.

[44] Díaz AV Walker M Webster JP. Reaching the World Health Organization elimination targets for schistosomiasis: the importance of a one health perspective. Phil Trans R Soc B. (2023) 378:20220274. 10.1098/rstb.2022.0274 37598697 PMC10440173

[45] Douchet P Gourbal B Loker ES Rey O. Schistosoma transmission: scaling-up competence from hosts to ecosystems. Trends Parasitol. (2023) 39:563–74. 10.1016/j.pt.2023.04.001 37120369 PMC10880732

[46] Vicente-Santos A Willink B Nowak K Civitello DJ Gillespie TR. Host-pathogen interactions under pressure: a review and meta-analysis of stress-mediated effects on disease dynamics. Ecol Lett. (2023) 1–18. 10.1111/ele.14319 37804128 PMC10874615

[47] Coates A. Research pre-empting parasite adaptation is key to sustainable disease management in aquaculture. Aquacult Environ Interact. (2023) 15:35–43. 10.3354/aei00451

[48] Hodžić A Dheilly NM Cabezas-Cruz A Berry D. The helminth holobiont: a multidimensional host–parasite–microbiota interaction. Trends Parasitol. (2023) 39:91–100. 10.1016/j.pt.2022.11.012 36503639

[49] Hector TE Gehman ALM King KC. Infection burdens and virulence under heat stress: ecological and evolutionary considerations. Phil Trans R Soc B. (2023) 378:20220018. 10.1098/rstb.2022.0018 36744570 PMC9900716

[50] Poglayen G Gelati A Scala A Naitana S Musella V Nocerino M. Do natural catastrophic events and exceptional climatic conditions also affect parasites? Parasitol. (2023) 15:1–9. 10.1017/S0031182023000471 37183698 PMC10801372

